
JOURNAL INFORMATION
==============================
NLM Title Abbreviation: ScientificWorldJournal
Journal ID: ScientificWorldJournal
NLM Journal ID: 101131163
Journal ID: TSWJ

The Scientific World Journal

ISSN: 2356-6140
EISSN: 1537-744X
Publisher: Wiley

ARTICLE INFORMATION
==============================
PMCID: PMC5956389
PMID: 15349538
DOI: 10.1100/tsw.2004.58
Article ID: 491269
Article version: 1
Subjects: Research Article

Physiology of Penile Erection

El-Sakka Ahmed I.
Lue Tom F.
Department of Urology, University of California School of Medicine, San Francisco, CA, USA
Academic Editor: Anthony Atala

Publication date: 2004
Electronic publication date: 2004 Jun 7
Volume: 4
First page: 128
Last page: 134
Copyright: Copyright © 2004 Ahmed I. El-Sakka and Tom F. Lue.
Copyright year: 2004
License: This is an open access article distributed under the Creative Commons Attribution License, which permits unrestricted use, distribution, and reproduction in any medium, provided the original work is properly cited.
License URL: https://creativecommons.org/licenses/by/3.0/

==============================
Article
*TheScientificWorldJOURNAL* (2004) 4 (S1), 128–134
ISSN 1537-744X; DOI 10.1100/tsw.2004.58

### Physiology of Penile Erection

Ahmed I. El-Sakka, M.D. and Tom F. Lue, M.D.
*Department of Urology, University of California School of Medicine, San Francisco, California*

**E-mail**: elsakka@ismailia.ie.eg.com

*Previously published in the Digital Urology Journal*

---

**DOMAIN**: urology

---

Erection is the final common pathway of the integrative synchronized action of psychological, neuronal, hormonal, vascular, and cavernous smooth muscle systems. In this short review we will discuss the advancement of knowledge that the last decade have witnessed.

### HEMODYNAMICS AND MECHANISM OF ERECTION AND DETUMESCENCE

### Corpora Cavernosa

The penile erectile tissue, specifically the cavernous smooth musculature and the smooth muscles of the arteriolar and arterial walls, plays a key role in the erectile process. In the flaccid state, these muscles are tonically contracted by the sympathetic discharge, and vasoconstrictors secreted by endothelium allowing only a small amount of arterial flow for nutritional purposes.

Sexual stimulation triggers release of neurotransmitters from the cavernous nerve terminals. This results in relaxation of these smooth muscles and the following events: (1) dilation of the arterioles and arteries by increased blood flow in both the diastolic and systolic phases; (2) trapping of the blood by the expanding sinusoids; (3) compression of the subtunical venular plexuses between the tunica albuginea and the peripheral sinusoids, reducing the venous outflow; (4) stretching of the tunica to its capacity, which encloses the emissary veins between the inner circular and the outer longitudinal layers and further decreases the venous outflow to a minimum; (5) an increase in intracavernous pressure (maintained at around 100 mm Hg), which raises the penis from the dependent position to the erect state (the full erection phase); and (6) a further pressure increase (to several hundred mm Hg) with contraction of the ischiocavernosus muscles (rigid erection phase.)

Three phases of detumescence have been reported in an animal study.[1] The first entails a transient intracorporeal pressure increase, indicating the beginning of smooth muscle contraction against a closed venous system. The second phase shows a slow pressure decrease, suggesting a slow reopening of the venous channels with resumption of the basal level of arterial flow. The third phase shows a fast pressure decrease with fully restored venous outflow capacity.

Erection thus involves sinusoidal relaxation, arterial dilation and venous compression.[2] The importance of smooth muscle relaxation has been demonstrated in animal and human studies.[3,4]

### Corpus Spongiosum and Glans Penis

The hemodynamics of the corpus spongiosum and glans penis are somewhat different from those of the corpora cavernosa. During erection, the arterial flow increases in a similar manner; however, the pressure in the corpus spongiosum and glans is only one-third to one-half that in the corpora cavernosa because the tunical covering (thin over the corpus spongiosum and virtually absent over the glans) ensures minimal venous occlusion.

### Smooth Muscle Physiology

Spontaneous contractile activity of cavernous smooth muscle has been recorded in vitro and in vivo studies. Yarnitsky et al.[5] found two types of electrical activity recorded from the corpus cavernosum: spontaneous and activity-induced. Field stimulation results in a decrease in tension and intracellular calcium at low frequencies and an increase in tension with increased intracellular calcium at high frequencies. In general, the response to pharmacologic agents correlates with the change in intracellular calcium: e.g. phenylephrine produces muscle contraction and an increase in intracellular calcium, while nitroprusside causes the opposite.

### NEUROANATOMY AND NEUROPHYSIOLOGY OF PENILE ERECTION

### Peripheral Pathways

The innervation of the penis is both autonomic (sympathetic and parasympathetic) and somatic (sensory and motor). From the neurons in the spinal cord and peripheral ganglia, the sympathetic and parasympathetic nerves merge to form the cavernous nerves, which enter the corpora cavernosa and corpus spongiosum to effect the neurovascular events during erection and detumescence. The somatic nerves are primarily responsible for sensation and the contraction of the bulbocavernosus and ischiocavernosus muscles.

### Autonomic Pathways

The sympathetic pathway originates from the eleventh thoracic to the second lumbar spinal segments and passes via the white rami to the sympathetic chain ganglia. Some fibers then travel via the lumbar splanchnic nerves to the inferior mesenteric and superior hypogastric plexuses, from which fibers travel in the hypogastric nerves to the pelvic plexus. In man, the $T_{10}$ to $T_{12}$ segments are most often the origin of the sympathetic fibers, and the chain ganglia cells projecting to the penis are located in the sacral and caudal ganglia.[6]

The parasympathetic pathway arises from neurons in the intermediolateral cell columns of the second, third and fourth sacral spinal cord segments. The preganglionic fibers pass in the pelvic nerves to the pelvic plexus, where they are joined by the sympathetic nerves from the superior hypogastric plexus.

The cavernous nerves are branches of the pelvic plexus that innervate the penis. The cavernous nerves are easily damaged during radical excision of the rectum, bladder and prostate. A clear understanding of the course of these nerves is essential to the prevention of iatrogenic erectile dysfunction.[7]

Stimulation of the pelvic plexus and the cavernous nerves induces erection, whereas stimulation of the hypogastric nerve or the sympathetic trunk causes detumescence. This clearly implies that the sacral parasympathetic input is responsible for tumescence, and the thoracolumbar sympathetic pathway for detumescence.

The cerebral impulses normally travel through sympathetic (inhibiting norepinephrine release), parasympathetic (releasing nitric oxide and acetylcholine), and somatic (releasing acetylcholine) pathways to produce a normal rigid erection. In patients with a sacral cord lesion, the cerebral impulse can still travel via the sympathetic pathway to inhibit norepinephrine release, and nitric oxide and acetylcholine can still be released via synapse with postganglionic parasympathetic and somatic neurons. Because the number of synapses between the thoracolumbar outflow and postganglionic parasympathetic and somatic neurons is less than the sacral outflow, the resulting erection will not be as strong.

### Somatic Pathways

The somatosensory pathway originates at the sensory receptors in the penile skin, glans, urethra and within the corpus cavernosum. In the human glans penis, there are numerous afferent terminations: free nerve endings and corpuscular receptors with a ratio of 10:1. The free nerve endings are derived from thin myelinated and unmyelinated C fibers and are unlike any other cutaneous area in the body.[8] The nerve fibers from the receptors converge to form bundles of the dorsal nerve of the penis, which joins other nerves to become the pudendal nerve. Activation of these sensory receptors sends messages of pain, temperature, and touch via the dorsal and pudendal nerves, spinal cord, and spinothalamic tract to the thalamus and sensory cortex for sensory perception. Recent evidences clearly demonstrate that the dorsal nerve is a mixed nerve with both somatic and autonomic components that enable it to regulate both erectile and ejaculatory function.[9,10] Onuf's nucleus in the second to fourth sacral spinal segments is the center of somatomotor penile innervation. These nerves travel in the sacral nerves to the pudendal nerve to innervate the ischiocavernosus and bulbocavernosus muscles. Contraction of the ischiocavernosus muscles produces the rigid erection phase. Rhythmic contraction of the bulbocavernosus muscle is necessary for ejaculation.

In summary, the structures above are responsible for the three types of erection: psychogenic, reflexogenic and nocturnal. Psychogenic erection is a result of audiovisual stimuli or fantasy. Impulses from the brain modulate the spinal erection centers (T11-L2 and S2-S4) to activate the erectile process. Reflexogenic erection is produced by tactile stimuli to the genital organs. The impulses reach the spinal erection centers; some then follow the ascending tract, resulting in sensory perception, while others activate the autonomic nuclei to send messages via the cavernous nerves to the penis to induce erection. This type of erection is preserved in patients with upper spinal cord injury. Nocturnal erection occurs mostly during rapid-eye-movement (REM) sleep. The mechanism is as yet unknown.

### Neurotransmitters

### *Peripheral Neurotransmitters*

#### *Flaccidity and Detumescence*

Adrenergic nerve fibers and receptors have been demonstrated in the cavernous trabeculae and surrounding the cavernous arteries, and norepinephrine has generally been accepted as the principal neurotransmitter to control penile flaccidity and detumescence.[11,12] Receptor binding studies have shown the number of a-adrenoceptors to be 10 times higher than the number of ß-adrenoceptors.[13]

Endothelin, a potent vasoconstrictor produced by the endothelial cells, has also been suggested to be a neurotransmitter for detumescence.[14,15] In addition, other vasoconstrictors such as thromboxane $A_2$, prostaglandin $F_{2a}$ and leukotrienes have also been proposed.[16,17]

### Erection

Acetylcholine is required for ganglionic transmission (by nicotinic receptors) and vascular smooth muscle relaxation (by muscarinic receptors). Cholinergic nerves have been demonstrated within the human cavernous smooth muscle and surrounding penile arteries, and ultrastructural examination has also identified terminals containing cholinergic vesicles in the same area.[18] It has been suggested that acetylcholine stimulates the release of NO from endothelial cells and thus contributes directly to smooth muscle relaxation during erection.[19]

Recent observations strongly suggest that NO released from nonadrenergic/noncholinergic (NANC) neurons increases the production of cyclic guanosine monophosphate (cGMP), which in turn relaxes the cavernous smooth muscle.[20] Kim et al[21] have also shown that the NO-mediated responses are progressively inhibited as a function of decreasing oxygen tension; reverting to normal oxygen tension restores endothelium-dependent and neurogenic relaxation. Currently, NO or an NO-like substance appears to be the most likely principal neurotransmitter causing penile erection.

### Nitric Oxide and cGMP in Relaxation of Smooth Muscle

Nitric oxide was first described in 1979 as a potent relaxant of peripheral vascular smooth muscle, with an action mediated by cGMP.[22] Subsequently, endothelium-derived relaxing factor was identified as NO or chemically unstable nitroso precursor.[23,24] Nitric oxide is synthesized from endogenous L-arginine by NO synthase (NOS) located in the vascular endothelium.[25] Nitric oxide may be synthesized and released as a neurotransmitter by the NANC neurons after their excitation by either electrical or chemical stimulation. In this regard Ignarro et al[26] have suggested that NO is highly labile; therefore, it cannot be stored as a preformed neurotransmitter. There are at least three distinct forms of NOS (neuronal, endothelial and macrophage).[27] The three forms of NOS display about 50% identity in amino acid sequence. The constitutive isoforms are generally regulated by Ca++-calmodulin, while inducible forms are not. Most of the isoforms require NADPH, tetrahydrobiopterin, FAD and FMN.

Other investigators believe that VIP may be one of the neurotrans-mitters responsible for erection. VIP-immunoreactive nerve fibers have been identified within the cavernous trabeculae and surrounding penile arteries, and neurostimulation-induced cavernous smooth muscle relaxation has been shown to be blocked by a VIP antagonist or anti-VIP serum.[28,29] Other potential candidates include calcitonin-gene-related peptide (CGRP)[30] peptide histidine methionine,[31] pituitary adenylate cyclase-activating polypeptide 32, and prostaglandins 33,34. Prostaglandin $E_1$ receptor density is reported to be lower in impotent men.[35] Although these neurotransmitters may participate in the erectile process, they are unlikely to play a major role. Nevertheless, because of the multiplicity of putative transmitters present in the corpus cavernosum and in perivascular nerves, further investigation is needed to elucidate the interactions between neurotransmitters and neuromodulators at the neuromuscular junction, and between the neural and endothelial control of vascular tone.[36]

### Central Neurotransmitters

A variety of neurotransmitters, including dopamine, norepinephrine and serotonin, have been identified in the medial preoptic area "MPOA" and paraventricular areas of hypothalamus.[37] It is suggested that dopaminergic and adrenergic receptors may promote sexual drive, and that serotonin receptors inhibit it.[38]

### PHARMACOLOGY OF PENILE ERECTION

### Intracavernous and Topical Drugs

The introduction of intracavernous injection of vasoactive drugs has revolutionized the diagnosis and treatment of erectile dysfunction. Most researchers agree that erection results from relaxation of the smooth muscle of the arterioles and the cavernous trabeculae, whereas detumescence is the result of contraction of these smooth muscles. Therefore, vasoactive drugs, injected intracavernously, can be used to induce erection (muscle relaxants) or detumescence (a-adrenergic agonists) through their effect on smooth muscle.

The mechanism of smooth muscle relaxation in response to various vasodilators is complex and is not completely understood, especially when a combination of drugs such as papaverine, phentolamine and prostaglandin $E_1$ is used.

Recently, several studies of the transdermal application of vasoactive drugs, including nitroglycerin cream or paste, minoxidil and stearyl-VIP,[39] have been reported. Transurethral application of vasoactive agents such as prostaglandin $E_1$ and $E_2$ has also shown promise.[40]

Inhibitors of phosphodiesterase, which normally degrades cGMP and cAMP, are an interesting group of compounds.[41] They comprise seven subtypes, and the specific cGMP inhibitor (type 5) (Sildenafil; Pfizer) has been shown to enhance penile erection when given orally. The success in oral drug therapy represents another major advance in the treatment of erectile dysfunction.

### Centrally Acting Drugs

Drugs that activate central adrenergic receptors have been reported to increase the libido of patients who take L-dopa, amphetamines and deprenyl. On the other hand, prazosin (an $a_1$-antagonist) and clonidine ($a_2$-agonist) suppress mating behavior, whereas yohimbine ($a_2$-antagonist) increases it.[42]

Dopamine agonists (apomorphine and pergolide) and dopamine uptake inhibitors (nomifensine and bupropion) have been reported to enhance sexual drive in patients.[43] In animal studies, selective activation of D2-dopaminergic receptors by the systemic administration of agonists such as apomorphine and quinelorane increases sexual behavior, and this effect can be counteracted by centrally acting antagonists.

Serotonin (5-hydroxytryptamine [5-HT]) is believed to be an inhibitory transmitter in the control of sexual drive.[44] Suppressed libido in patients taking fenfluramine, a 5-HT-releasing agent, and elevated libido in patients taking buspirone, a 5-HT neuron suppressor, have been reported.[45] Clinically, trazodone has been reported to enhance nocturnal penile erection and cause priapism in men.[46]

### REFERENCES

1. Bosch, R. J., Benard, F., Aboseif, S. R., Stief, C, G., Lue, T. F., and Tanagho, E. A.: Penile detumescence: characterization of three phases. J. Urol., 146: 867, 1991.
2. Lue, T. F., Takamura, T., Schmidt, R. A., Palubinskas, A. J., and Tanagho, E. A.: Hemodynamics of erection in the monkey. J. Urol., 130:1237, 1983.
3. Ignarro, L. J., Bush, P. A., Buga, G. M., Wood, K. S., Fukuto, J. M., and Rajfer, J.: Nitric oxide and cyclic GMP formation upon electrical field stimulation cause relaxation of corpus cavernosum smooth muscle. Biochem. Biophys. Res. Comm. 170: 843, 1990.
4. Saenz de Tejada, I., Moroukian, P., Tessier, J., Kim, J. J., Goldstein, I., and Frohrib, D.: Trabecular smooth muscle modulates the capacitor function of the penis. Studies on a rabbit model. Am. J. Physiol. 260:H1590. 1991.
5. Yarnitsky, D., Sprecher, E., Barilan, Y., and Vardi, Y.: Corpus cavernosum electromyogram: spontaneous and evoked electrical activities. J. Urol., 153:653, 1995.
6. DeGroat, W. C., and Booth, A. M.: Neural control of penile erection. In: Maggi CA, ed. The Autonomic Nervous System. Nervous Control Of The Urogenital System. Chap 13. London: Harwood. pp 465-513. 1993.
7. Walsh, P. C., Brendler, C. B., Chang, T., Marshall, F. F., Mostwin, J. I., Stutzman, R., and Schlegel, P. N.: Preservation of sexual function in men during radical pelvic surgery. M. Med. J. 39:389, 1990.

8. Halata, Z., and Munger, B. L.: The neuroanatomical basis for the protopathic sensibility of the human glans penis. Brain Res. 371:205 1986.
9. Burnett, A. L., Tillman, S. L., Chang, T. S., and Epstein, J. I., Lowenstein, C. J., Bredt, D. S., Snyder, S. H., and Walsh, P. C.: Immunohistochemical localization of nitric oxide synthase in the autonomic innervation of the human penis. J. Urol., 150:73 1993.
10. Carrier, S., Zvara, P., Nunes, L., Kour, N. W., Rehman, J., and Lue T. F.: Regeneration of nitric oxide synthase-containing nerves after cavernous nerve neurotomy in the rat. J. Urol., 153:1722, 1995.
11. Hedlund, H., and Andersson, K.: Comparison of the responses to drugs acting on adrenoreceptors and muscarinic receptors in human isolated corpus cavernosum and cavernous artery. J. Auton. Pharmacol. 5:81, 1985.
12. Diederich, W., Stief, C. G., Lue, T. F., and Tanagho, E. A.: Norepinephrine involvement in penile detumescence. J. Urol., 143:1264, 1990.
13. Levin, R. M., and Wein, A. J.: Adrenergic alpha receptors outnumber beta receptors in human penile corpus cavernosum. Invest. Urol., 18:225 1980.
14. Holmquist, F., Andersson, K., and Hedlund H. : Actions of endothelin on isolated corpus cavernosum from rabbit and man. Acta Physiol. Scand. 139:113, 1990.
15. Saenz de Tejada, I., Carson, M. P., de las Morenas, A., Goldstein, I., and Traish, A. M.: Endothelin: localization, synthesis, activity, and receptor types in human penile corpus cavernosum. Am. J. Physiol. X 261:H1078, 1990.
16. Hedlund, H., Andersson, K., Fovaeus, M., Holmquist, F., and Uski, T.: Characterization of contraction-mediating prostanoid receptors in human penile erectile tissues. J. Urol., 141:182, 1989.
17. Azadzoi, K. M., Kim, N., Brown, M. L., Goldstein, I., Cohen, R. A., and Saenz de Tejada, I.: Endothelium-derived nitric oxide and cyclooxygenase products modulate corpus cavernosum smooth muscle tone. J. Urol., 147:220, 1992.
18. Steers, W. D., McConnell, J., and Benson, G.: Anatomical localization and some pharmacological effects of vasoactive intestinal polypeptide in human and monkey corpus cavernosum. J. Urol., 132:1048, 1984.
19. Saenz de Tejada, I., Goldstein, I., Azadzoi, K., Krane, R. J., and Cohen, R., A.: Impaired neurogenic and endothelium-mediated relaxation of penile smooth muscle from diabetic men with impotence. N.E.J.M. 320:1025, 1989.
20. Ignarro, L.,J, Bush, P. A., Buga, G. M., Wood, K. S., Fukuto, J. M., and Rajfer J. Nitric oxide and cyclic GMP formation upon electrical field stimulation cause relaxation of corpus cavernosum smooth muscle. Biochem. Biophys. Res. Comm ; 170:843, 1990.
21. Kim, N., Vardi, Y., Padma-Nathan, H., Daley, J., Goldstein, I., and Saenz de Tejada, I.: Oxygen tension regulates the nitric oxide pathway. Physiological role in penile erection. J. Clin. Invest. 91:437, 1993.
22. Gruetter, C. A., Barry, B. K., McNamara, D. B., Gruetter, D. Y., Kadowitz, P. J., and Ignarro, L. J.: Relaxation of bovine coronary artery and activation of coronary arterial guanylate cyclase by nitric oxide, nitroprusside and a carcinogenic nitrosoamine. J. Cyclic Nucleotide Res. 5:211, 1979.
23. Ignarro, L. J., Buga, G. M., Wood, K. S., Byrns, R. E., and Chaudihuri, G.: Endothelium derived relaxing factor produced and released from artery and vein is nitric oxide. Proc. Natl. Acad. Sci.USA 84:9265, 1987.
24. Palmer, R. M. J., Ferrige, A. G., and Moncada, S.: Nitric oxide release accounts for the biological activity of endothelium-derived relaxing factor. Nature 327:524, 1987.
25. Palmer, R. M. J., Ashton, D. S., and Moncada, S.: Vascular endothelial cells synthesize nitric oxide from L-arginine. Nature 333:664, 1988.
26. Ignarro, L. J., Bush, P. S., Buga, G. M., and Rajfer, J.: Neurotransmitter identity in doubt. Nature 347:131.1990b.
27. Lowenstein, C. J., Glatt, C. S., Bredt, D. S., and Snyder, S. H.: Cloned and expressed macrophage nitric oxide synthase contrasts with the brain enzyme. Proc. Natl. Acad .Sci. USA 89:6711, 1992.
28. Ottesen, B., Wagner, G., Virag, R., and Fahrenkrug, J.: Penile erection: possible role for vasoactive intestinal polypeptide as a neurotransmitter. Br. Med. J., 288:9, 1984.
29. Kim, Y. C., Kim, J. H. , Davies, M. G., Hagen, P. O., and Carson, C. C.: Modulation of vasoactive intestinal polypeptide (VIP)-mediated relaxation by nitric oxide and prostanoids in the rabbit corpus cavernosum. J. Urol., 153:807, 1995.
30. Stief, C. G., Benard, F., Bosch, R. J., Aboseif, S. R., Lue, T. F., and Tanagho, E.: A. A possible role of calcitonin-gene-related peptide in the regulation of the smooth muscle tone of the bladder and penis. J. Urol., 143:392, 1990.
31. Kirkeby, H. J., Fahrenkrug, J., Holmquist, F.,and Ottesen, B..: Vasoactive intestinal polypeptide (VIP) and peptide histidine methionine (PHM) in human penile corpus cavernosum tissue and circumflex veins: localization and in vitro effects. Eur. J. Clin. Invest.22:24, 1992.
32. Hedlund, P., Alm, P., Hedlund, H., Larsson, B., and Andersson, K. E.: Localization and effects of pituitary adenylate cyclase-activating polypeptide (PACAP) in human penile erectile tissue. Acta Physiologica Scandinavica 150:103, 1994.
33. Saenz de Tejada, I., Kim, N., Lagan, I., Krane, RJ. , and Goldstein, I.: Regulation of adrenergic activity in penile corpus cavernosum. J. Urol., 142:1117, 1989.
34. Adaikan, P. G., and Ratnam, S. S.: Pharmacology of penile erection in humans. Cardiovas. Intervent. Radiol. 11:191, 1988.
35. Aboseif, S., Riemer, R. K., Stackl, W., Lue,T. F., and Tanagho, E. A.: Quantification of prostaglandin E1 receptors in cavernous tissue of men, monkeys and dogs. Urol. Int. 50: 226-450, 1993.

36. Andersson, K-E., and Holmquist, F.: Regulation of tone in penile cavernous smooth muscle. Established concepts and new findings. World J. Urol., 12:249, 1994.
37. Simerly, R. B., and Swanson, L. W.: Projections of the medial preoptic nucleus: a Phaseolus vulgaris leucoagglutinin anterograde tract-tracing in the rat. J. Comp. Neurol. 270:209, 1988.
38. Foreman, M. M., and Wernicke, J.F.: Approaches for the development of oral drug therapies for erectile dysfunction. Semin. Urol. 8:107, 1990.
39. Gozes, I., Reshef, A., Salah, D., Rubinraut, S., and Fridkin., M.: Stearyl-norleucine-vasoactive intestinal peptide (VIP): a novel VIP analog for noninvasive impotence treatment. Endocrinology 134:2121, 1994.
40. Wolfson, B., Pickett, S., Scott, N. E., DeKernion, J. B., and Rajfer J.: Intraurethral prostaglandin E-2 cream: a possible alternative treatment for erectile dysfunction. Urology 42:73, 1993.
41. Sparwasser, C., Drescher, P., Will, J. A., and Madsen, P. O.: Smooth muscle tone regulation in rabbit cavernosal and spongiosal tissue by cyclic AMP-and cyclic GMP-dependent mechanisms. J. Urol., 152:2159, 1994.
42. Clark, J. T., Smith, E. R., and Davidson, J. M.: Evidence for modulation of sexual behavior by alpha-adrenoceptors in male rats. Neuroendocrinology 41:36, 1985.
43. Jeanty, P., Van den, Kerchove, M., Lowenthal, A., De Bruyne, H.: Pergolide therapy in Parkinson's disease. J. Neurol., 231:148, 1984.
44. Foreman, M. M., Hall, J. L., and Love, R. L.: The role of the 5-HT2 receptor in the regulation of sexual performance of male rats. Life Sci. 45:1263, 1989.
45. Buffum, J.: Pharmacosexology: the effects of drugs on sexual function. A review. J. Psychoactive Drugs 14:5, 1982.
46. Saenz de Tejada, I., Ware, J. C., Blanco, R., Pittard, J.T., Nadig, P. W., Azadzoi K. M., Krane, R. J., and Goldstein, I.: Pathophysiology of prolonged penile erection associated with trazodone use. J. Urol., 145:60, 1991.

**This article should be referenced as follows:**

El-Sakka, A.I. and Lye, T.F. (2004) Physiology of penile erection. *TheScientificWorldJOURNAL* **4 (S1)**, 128–134.

**Handling Editor:**

Anthony Atala, Principle Editor for *Urology* — a domain of *TheScientificWorldJOURNAL*.
